# A QUALITATIVE ANALYSIS OF PERCEIVED BEREAVEMENT NEEDS OF COMMUNITY-DWELLING OLDER ADULTS

**DOI:** 10.1093/geroni/igae098.3336

**Published:** 2024-12-31

**Authors:** Nicole Praska, Marcus Chur, Caroline Collins-Pisano, Rachel Weiskittle

**Affiliations:** University of Colorado, Colorado Springs, Colorado Springs, Colorado, United States; University of Colorado Colorado Springs, Colorado Springs, Colorado, United States; University of Colorado, Colorado Springs, Colorado Springs, Colorado, United States; University of Colorado, Colorado Springs, Colorado Springs, Colorado, United States

## Abstract

This paper presentation will discuss the qualitative findings of a city-wide community needs assessment on available grief support with particular emphasis on service needs for older adults. The purposive sampling of this survey targeted local professionals who frequently interact with bereaved individuals in their work, such as mental health providers, funeral directors, faith leaders, and healthcare providers. Survey items asked participants to identify local grief support demand, engagement, barriers, and interests. Of the 141 participants who completed the survey, 80 (57%) provided free response answers eligible for qualitative analysis. Themes were extracted using manifest coding across three raters. Major themes on older adult bereavement include: perceived gaps in available services, service format suggestions (e.g., virtual vs. in-person, faith-based vs. secular), and suggestions for the creation of older adult grief support based on type of loss (e.g., pet loss, kinshiplessness), and perceived educational interest, such as substance use and emotions in grief. In this presentation, qualitative methodology of the study, as well as its resulting themes and subthemes, will be further described to offer insight into participant perspectives on service needs for older adult bereavement.

